# Gadd45a levels in human breast cancer are hormone receptor dependent

**DOI:** 10.1186/1479-5876-11-131

**Published:** 2013-05-24

**Authors:** Jennifer S Tront, Alliric Willis, Yajue Huang, Barbara Hoffman, Dan A Liebermann

**Affiliations:** 1 Fels Institute for Cancer Research and Molecular Biology, Philadelphia, USA; 2 Department of Surgery, Philadelphia, USA; 3 Department of Pathology and Laboratory Medicine, Philadelphia, USA; 4 Department of Biochemistry, Temple University School of Medicine, Philadelphia, PA 19140, USA

**Keywords:** Gadd45a, Breast cancer, Hormone receptor

## Abstract

**Background:**

Gadd45a is a member of the Gadd45 family of genes that are known stress sensors. Gadd45a has been shown to serve as an effector in oncogenic stress in breast carcinogenesis in murine models. The present study was aimed at clarifying the expression of Gadd45a in human breast cancer and its correlation with clinicopathologic features.

**Methods:**

The expression levels of Gadd45a in breast tissue samples of female breast surgery cases were examined by immunohistochemistry (IHC) using a Gadd45a antibody. Percent staining was determined and statistical analyses were applied to determine prognostic correlations.

**Results:**

56 female breast surgery cases were studied: Normal (11), Luminal A (9), Luminal B (11), HER2+ (10), Triple Negative (15). There was a highly significant difference in percent Gadd45a staining between groups [Mean]: Normal 16.3%; Luminal A 65.3%; Luminal B 80.7%; HER2+ 40.5%; TN 32%, P < 0.001, ANOVA. Gadd45a IHC levels for Normal cases found 82% negative/low. Luminal A breast cancer cases were found to be 67% high. Luminal B breast cancers were 100% high. Her2+ cases were 50% negative/low. Triple Negative cases were 67% negative/low. This difference in distribution of Gadd45a levels across breast cancer receptor subtypes was significant, P = 0.0009.

**Conclusions:**

Gadd45a levels are significantly associated with hormone receptor status in human breast cancer. Normal breast tissue displays low Gadd45a levels. High Gadd45a levels are associated with Luminal A and Luminal B subtypes. Absence of hormone receptors in Triple Negative subtype is associated with Negative/Low levels of Gadd45a. Further studies are indicated to elucidate the role of Gadd45a in breast cancer as a potential prognosticator or target for treatment.

## Background

In the United States, breast cancer is the most common non-skin cancer among women, with an estimated 230,000 cases diagnosed in 2012 [1,2]. It is also one of the leading causes of cancer death among women of all races and Hispanic origin populations. However, a full understanding of the molecular mechanisms that contribute to the malignant growth of breast cancer cells remains unclear. Therefore, understanding the complete biology of breast cancer is an important aspect for identifying therapeutic targets.

Gadd45a is a member of the Gadd45 family of genes that are known stress sensors, which modulates the cellular response to a variety of stress conditions, including genotoxic and oncogenic stress [3-6]. The complex role of stress sensors in monitoring tumor development is not fully understood, the best example being the multiple functions attributed to p53 in tumor development and suppression [7,8]. Gadd45a is a transcriptional target for tumor suppressors p53 and BRCA1, whose loss of function play key roles in cancer development, including breast tumorigenesis [7,8].

In murine models, Gadd45a functions to either promote or suppress breast tumor development via engagement of different signaling pathways depending on the molecular nature of the activated oncogene [9,10]. Although data suggests that Gadd45a may have a function in modulating human breast cancer, thus far there have been no reports on whether the expression of Gadd45a is correlated with any clinicopathological characteristics.

The present study was aimed at clarifying the expression of Gadd45a in human breast cancer and correlation of its expression with clinicopathologic features, such as hormone receptor status. In this study, we report for the first time the expression pattern of Gadd45a in breast cancer. We found that the expression levels of Gadd45a are hormone receptor dependent. Our results suggest that Gadd45a may play a role in breast cancer pathogenesis and could be used as a biomarker for breast cancer prognosis.

## Methods

### Tissue samples and patient clinical information

A total of 56 formalin-fixed paraffin embedded tissue samples were analyzed in this study, 45 primary breast carcinomas and 11 normal breast tissue. Normal samples were obtained from patients who had undergone reduction mammaplasty surgery at Temple University Hospital. Breast carcinoma samples were obtained from patients who had undergone surgery and were histopathologically and clinically diagnosed from 2005 to 2011. Clinical diagnosis and staging were determined according to the WHO tumor classification (International Classification of Diseases in Oncology) [11] and the AJCC Cancer Staging Manual (7th Edition) [12]. The histopatholgical grading was done according to the modified Bloom and Richardson system [11]. All work was performed under a Temple University Institutional Review Board approved protocol (Protocol Number 12485). Patient consent was obtain according to guidelines. Table 1 summarizes clinical and pathological information for all patients.

**Table 1:** Clinicopathological characteristics

		**Number**	**Percentage**
Cases			
	Normal	11	
	Cancer	45	
Grade			
	I	11	24%
	II	14	31%
	III	20	45%
Estrogen receptor			
	Positive	20	44%
	Negative	25	56%
Progesterone receptor			
	Positive	19	42%
	Negative	26	58%
HEr2/Neu			
	Positive	21	47%
	Negative	24	53%
Age			
	≤45	9	20%
	>45	36	80%
T Classification			
	T1	16	36%
	T2	19	42%
	T3	8	18%
	T4	2	4%
N Classification			
	N0	21	47%
	N1	16	36%
	N2	5	11%
	N3	3	6%

### Immunohistochemistry

Immunohistochemical analysis was performed on 56 human breast tissues. To summarize, paraffin-embedded tissue slides were deparaffinized, rehydrated and subjected to antigen unmasking by sodium citrate (pH 6.0) for 30 minutes at a sub-boiling temperature. Endogenous peroxidase activity was blocked by incubation in 3% hydrogen peroxide for 10 minutes. Sections were blocked with 5% serum for one hour at room temperature, followed by incubation with primary antibody overnight at 4°C. (Gadd45A - Abcam (ab7664)). For a negative control, the anti–Gadd45a antibody was replaced with normal goat serum. Sections were incubated with a peroxidase-conjugated secondary antibody for 30 min at room temperature, followed by treatment with ABC reagent (Vector Laboratories) for 30 min. Sections were stained with 3,3′-diaminobenzidine substrate and counterstained with hematoxylin, dehydrated and mounted in Permount (Fisher Scientific). Slides were prepared in triplicate. The degree of immunostaining was reviewed independently by three observers, including an attending pathologist. Percent positive stainings for each specimen were averaged and categorized as: Negative/Low (0- < 30%, Neg), Medium (30–60%, Med), or High (>60%).

### Statistical analysis

All statistical analyses were carried out using GraphPad Prism Statistical Software. ANOVA was used to analyze the correlation between the mean percent Gadd45a staining and the clinicopathological characteristics. Chi Square Analysis was performed on percent staining categories. P < 0.05 in all cases was considered statistically significant.

## Results

### Elevated expression of Gadd45a in primary breast tumors

**Figure 1:**
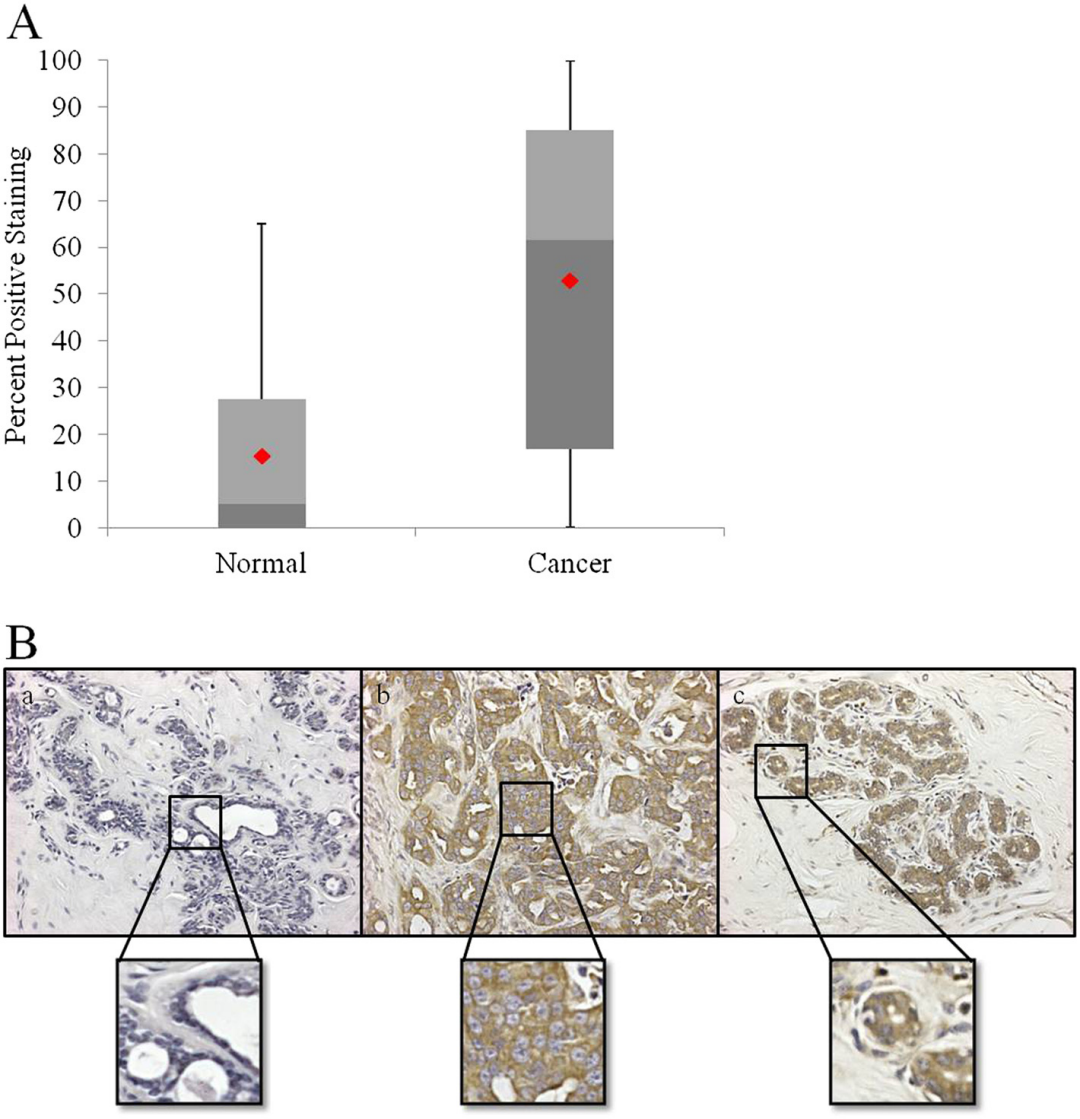
**Gadd45a expression levels are elevated in human breast cancer samples compared to normal tissue.** (**A**) Box and Whisker Plots, graphically depicting Gadd45a expression in human breast samples, demonstrate elevated levels of Gadd45a expression in carcinoma samples compared to normal tissue samples. Statistical significance was achieved between differences in mean percent staining between the two groups (P < 0.001, t-test). (**B**) Representative images from immunohistochemical analysis of Gadd45a on (**a**) normal breast tissue, (**b**) breast cancer tissue and (**c**) normal epithelial cells adjacent to cancer cells.

To investigate Gadd45a expression in human breast cancer, 45 previously characterized human female mammary carcinoma samples and 11 normal mammary samples were evaluated for Gadd45a expression by immunohistochemistry analysis with an antibody against Gadd45a. We found that Gadd45a was significantly overexpressed in breast cancer tissues compared to normal tissues obtained from patients who underwent reduction mammaplasty. (P < 0.001, Figure 1A). High levels of strong cytoplasmic staining of Gadd45a were present in areas containing cancer cells of the primary breast tumors. In addition, strong cytoplasmic staining of Gadd45a was also detected in adjacent noncancerous epithelial cells (Figure 1B). Taken together, these observations demonstrate that high levels of Gadd45a expression are correlated with the development of primary breast carcinoma.

#### Gadd45a Expression is elevated in luminal A and luminal B breast cancers

**Figure 2:**
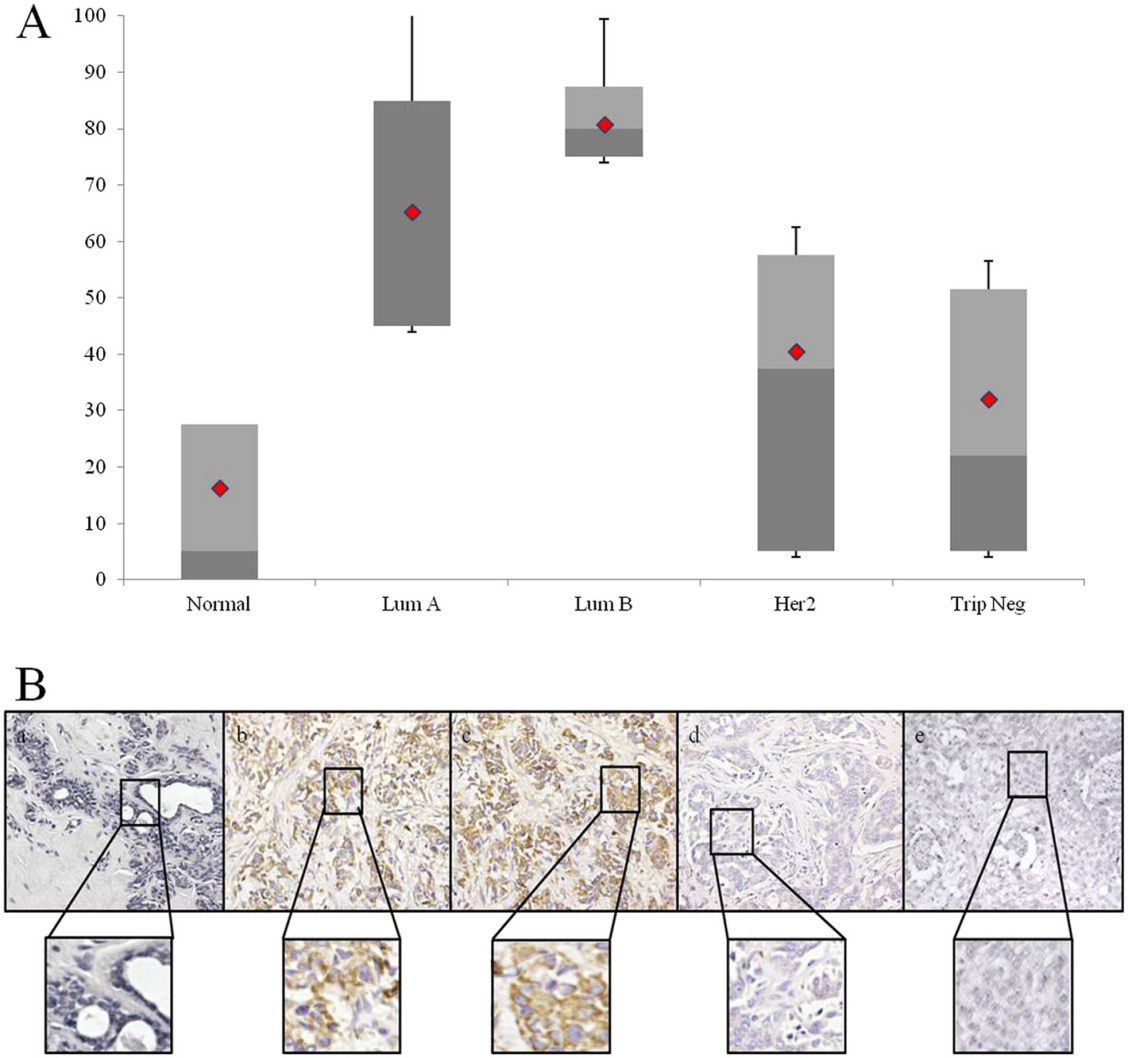
**Gadd45A expression levels in human breast cancer samples are dependent on the hormone receptor subtype.** (**A**) Box and Whisker Plots graphically depict the expression pattern of Gadd45a in the subtypes of human breast cancer. There was a highly significant difference in percent Gadd45a staining between groups: Normal 16.3%; Luminal A 65.3%; Luminal B 85.7%; HER2+ 40.5%; TN 31.2%, P < 0.001, ANOVA. (**B**) Representative images from immunohistochemical analysis of Gadd45a of Normal (**a**), Luminal A (**b**), Luminal B (**c**), Her2+ (**d**) and Triple Negative (**e**) breast tissue. Immunostaining was reviewed and scored by three independent observes. Percent positive stainings for each specimen were averaged and categorized as: Negative (0- < 10%, Neg), Low (10–<40%), Medium (40–<70%, Med), or High (≥70%).

In order to assess if Gadd45a expression levels correlated with any of breast cancer subtypes, the 56 breast surgery cases were grouped as follows: Normal (11, benign mammoplasty), Luminal A (9, ER+, PR+, HER2-; LumA), Luminal B (11, ER+, PR+, HER2+), HER2+ (10, ER-, PR-, HER2+), and Triple Negative (15, ER-, PR-, HER2-; TN ). There was a highly significant difference in percent Gadd45a staining between groups as measured by mean percent staining: Normal 16.3%; LumA 65.3%; LumB 80.7%; HER2+ 40.5%; TN 32% (Figure 2A). Figure 2B illustrates high levels of Gadd45a in Luminal A and Luminal B breast cancers. However, in Her2+/Neu and Triple Negative breast cancers, the levels of Gadd45a were low, similar to the levels observed in normal breast tissue. The observed differences in Gadd45a expression within the various breast cancer subtypes was statistically significant (P < 0.001, ANOVA).

The levels of Gadd45a expression, as calculated by percent staining for each specimen, were averaged and categorized as: Negative Low (<30%), Medium (31–60%, Med), or High (>60%). As seen in Table 2, Gadd45a expression levels for Normal cases found 81% low. Luminal A breast cancer cases were found to be 67% high. Luminal B breast cancers were 100% high. Her2+/Neu cases were 50% low, 20% med, 30% high. Triple Negative cases were 67% low. The difference in distribution of Gadd45a levels across breast cancer receptor subtypes was significant (P = 0.0009, Chi Squared test). Taken together, these data provide evidence that Gadd45a expression in breast cancer is elevated in Luminal A and Luminal B human breast cancer.

**Table 2:** Chi square analysis of gadd45a expression levels between breast cancer subtypes

	**Normal**	**Luminal A**	**Luminal B**	**Her2/Neu**	**Trip Neg**
Neg/Low <30	9	2	0	5	10
Medium 31–60	1	1	0	2	1
High >60	1	6	11	3	4

Chi Square P = 0.0009.

### Gadd45a Expression correlates with hormone receptor status

Statistical analyses were performed to examine the correlation between Gadd45a expression levels and various clinicopatholgical characteristics of breast cancer. No correlation was found between Gadd45a expression levels and cancer grade, patient age, Her2/Neu receptor status, T classification and N classification. However, as seen in Table 3, the expression of Gadd45a strongly correlated with ER/PR receptor status (P <0.001, Chi Squared test). Together, these data provide evidence that Gadd45a expression is Estrogen and/or Progesterone hormone receptor dependent.

**Table 3:** Chi square analysis of gadd45a expression levels between receptor status groups

	**Mean**	**Std**	**Pop size**
ER+/PR+	73.8	23.40625	20
ER+/PR-	35.4	35.19233	24
			*1 patient eliminated because ER/PR status were nonconcordant
	P < 0.001		
Her2/Neu+	61.57143	33.84313	21
Her2/Neu-	44.5	36.32343	24

## Discussion

We demonstrate for the first time that the expression levels of Gadd45a are hormone receptor status dependent, where high levels of Gadd45a correlate with ER+/PR + breast cancers. Gadd45a protein levels are elevated in human breast carcinoma, demonstrated by immunohistochemical staining. Our data presented here provides evidence that elevated expression of Gadd45a correlates with ER+/PR+, Luminal A and Luminal B breast cancers, whereas low expression of Gadd45a correlates with ER-/PR-, Her2+ and Triple Negative breast cancers. Taken together, our work provides evidence that Gadd45a may play a role in breast cancer pathogenesis and may represent a novel prognostic factor for breast cancer.

In the sample population from this study, the estrogen receptor and progesterone receptor status were concordant in all but one case, where the patient was ER+/PR-. Therefore, we were unable to discern whether Gadd45a expression correlates with estrogen receptor alone, progesterone receptor alone or in unison with both estrogen and progesterone receptor. Experiments are underway to address this question.

In addition to strong cytoplasmic staining of tumor cells, we find strong cytoplasmic staining in adjacent noncancerous epithelial cells. Knowing that Gadd45a is a stress sensor, playing a role in the cellular response to oncogenic stress, this finding supports the notion that Gadd45a may modulate the cellular response to oncogenic stress in surrounding noncancerous cells in human breast tissue thus playing a role in breast cancer pathogenesis. Future experiments are planned to further investigate the role Gadd45a plays in modulating the response of normal breast epithelial cells to adjacent cancerous cells.

Previously published studies have shown that Gadd45a plays a role in murine breast carcinoma [9,10]. Gadd45a functions as a tumor suppressor in Ras-driven breast tumorigenesis by increasing JNK-mediated apoptosis and p38-mediated senescence. In contrast, Gadd45a functions to promote Myc-driven breast cancer by negatively regulating MMP10 via GSK3B/B-catenin signaling, resulting in increased tumor vascularization and growth. Therefore, we choose to examine the expression patterns of these known Gadd45a effector proteins to provide insight into the functional role of Gadd45a in human breast cancer. Correlations between expression levels of Gadd45a and expression levels of activated JNK, p38, MMP10, GSK3B and B-catenin were examined. However, no correlations were found. Thus, additional experiments must be carried out to elucidate the molecular mechanism of Gadd45a modulation of human breast cancer.

A search for Gadd45a on public cancer microarray databases fails to provide statistically significant data to support our findings in this report that Gadd45a expression is elevated based on hormone receptor status. However, many microarray datasets do not allow for grouping based on clinicopatholgical characteristic parameters while analyzing the data. For those datasets that do allow for segregation based on those features, we believe that the discrepancy may be due to the fact that Gadd45a may not be regulated solely at the transcription level, but rather regulated at various levels.

In conclusion, Gadd45a levels in human breast cancer are significantly associated with hormone receptor status. Normal breast tissue has low levels of Gadd45a. Gadd45a levels are elevated in Luminal A subtypes, highest in Luminal B subtypes, lower in HER + subtypes and lowest in Triple Negative subtypes. In combination with other biomarkers of breast cancer, Gadd45a expression status may be useful for determining which therapeutic strategies against breast cancer are employed. Moreover, investigation of mechanisms that regulate Gadd45a expression during breast carcinogenesis and a more complete understanding of the role Gadd45a expression and signaling play in breast cancer pathogenesis may provide novel therapeutic targets.

## Conclusions

Gadd45a levels are significantly associated with hormone receptor status in human breast cancer. Normal breast tissue displays low Gadd45a levels. High Gadd45a levels are associated with Luminal A and Luminal B subtypes. Moderate levels of Gadd45a are associated with Her2 subtype samples. Absence of hormone receptors in Triple Negative subtype is associated with Negative/Low levels of Gadd45a. Our results suggest that Gadd45a may play a role in breast cancer pathogenesis and could be used as a biomarker for breast cancer prognosis.

## Abbreviations

Gadd45a: Growth arrest and dna damage 45 alpha; Her2: Human epidermal growth factor receptor 2; ER: Estrogen receptor; PR: Progesterone receptor; TN: Triple negative; Lum A: Luminal A; Lum B: Luminal B.

## Competing interests

There are no competing interests in regards to this manuscript.

## Authors’ contributions

JST participated in the conception and study design, carried out the experiments, performed statistical analysis and drafted the manuscript. AW carried out some immunostainings and performed statistical analysis. YH read all samples for immunostainings and provided expertise in pathology. BH participated in the conception and design of the study and reviewed all data. DAL conceived of the study, participated in the experimental design and reviewed all data. All authors read and approved the final manuscript.
